# From Evidence to Impact: Implementation Pathways for Elder Mistreatment Initiatives

**DOI:** 10.1093/geroni/igaf122.086

**Published:** 2025-12-31

**Authors:** Kristin Lees Haggerty, Tony Rosen

## Abstract

This symposium explores implementation science-informed approaches to elder mistreatment (EM) screening and intervention across healthcare contexts. The five presentations highlight complementary strategies with common implementation themes: adaptation of evidence-based tools for specific settings, addressing clinician barriers, and leveraging electronic health records. The first presentation demonstrates how integrating an EM protocol into emergency department electronic records increased screening rates from 2% to 85%, illustrating the power of workflow integration. The DETECT-RPC Project adapts a validated screening tool for home-based primary care, using the Theory of Planned Behavior to address clinician uncertainty about EM recognition and reporting requirements through targeted training interventions. The University of Colorado’s VESPA program showcases iterative adaptation of an elder abuse consultation service in response to implementation challenges. Their theory of change—that specialized teams offload complex and morally distressing cognitive work from medical providers—drove adaptive strategies as consult volumes outpaced resources, including developing a stepped care model and prioritizing complex cases. The Montana RISE-APS project represents a novel implementation approach, embedding the RISE intervention within Adult Protective Services (APS) rather than partnering externally. Using the Consolidated Framework for Implementation Research, this adaptation identifies contextual barriers and facilitators to implementation within APS. Finally, the Veterans Health Administration demonstrates system-wide implementation of standardized EM electronic tools, highlighting how researcher-clinician partnerships facilitate national dissemination in complex healthcare environments. Together, these presentations illustrate critical implementation science principles for EM interventions: context-sensitive adaptation, addressing workforce barriers, leveraging existing systems, and creating sustainable models that respond to real-world constraints while maintaining intervention fidelity.

